# Coexpression of GM-CSF and antigen in DNA prime-adenoviral vector boost immunization enhances polyfunctional CD8+ T cell responses, whereas expression of GM-CSF antigen fusion protein induces autoimmunity

**DOI:** 10.1186/1471-2172-9-13

**Published:** 2008-04-11

**Authors:** Matthias Tenbusch, Seraphin Kuate, Bettina Tippler, Nicole Gerlach, Simone Schimmer, Ulf Dittmer, Klaus Überla

**Affiliations:** 1 Department of Molecular and Medical Virology, Ruhr-University Bochum, 44801 Bochum, Germany; 2 Institute of Virology, University of Duisburg-Essen, 45122 Essen, Germany; 3 Immune Biology of Retroviral infections, VB, CCR, NCI, NIH, Bethesda, USA

## Abstract

**Background:**

Granulocyte-macrophage colony-stimulating factor (GM-CSF) has shown promising results as a cytokine adjuvant for antiviral vaccines and in various models of tumor gene therapy. To explore whether the targeting of antigens to GM-CSF receptors on antigen-presenting cells enhances antigen-specific CD8 T-cell responses, fusion proteins of GM-CSF and ovalbumin (OVA) were expressed by DNA and adenoviral vector vaccines. In addition, bicistronic vectors allowing independent expression of the antigen and the cytokine were tested in parallel.

**Results:**

*In vitro*, the GM-CSF ovalbumin fusion protein (GM-OVA) led to the better stimulation of OVA-specific CD8+ T cells by antigen-presenting cells than OVA and GM-CSF given as two separate proteins. However, prime-boost immunizations of mice with DNA and adenoviral vector vaccines encoding GM-OVA suppressed CD8+ T-cell responses to OVA. OVA-specific IgG2a antibody levels were also reduced, while the IgG1 antibody response was enhanced. Suppression of CD8+ T cell responses by GM-OVA vaccines was associated with the induction of neutralizing antibodies to GM-CSF. In contrast, the coexpression of GM-CSF and antigens in DNA prime adenoviral boost immunizations led to a striking expansion of polyfunctional OVA-specific CD8+ T cells without the induction of autoantibodies.

**Conclusion:**

The induction of autoantibodies suggests a general note of caution regarding the use of highly immunogenic viral vector vaccines encoding fusion proteins between antigens and host proteins. In contrast, the expansion of polyfunctional OVA-specific CD8+ T cells after immunizations with bicistronic vectors further support a potential application of GM-CSF as an adjuvant for heterologous prime-boost regimens with genetic vaccines. Since DNA prime adenoviral vector boost regimenes are presently considered as one of the most efficient ways to induce CD8+ T cell responses in mice, non-human primates and humans, further enhancement of this response by GM-CSF is a striking observation.

## Background

The induction of strong CTL responses by prophylactic and therapeutic vaccines is considered necessary for the control of chronic viral infections and cancer [1-3]. Genetic vaccines seem to be promising tools, since the expression of antigen by the vaccinee leads to improved MHC-I restricted cellular immune responses. DNA vaccines have been shown to elicit CTL, T helper and antibody responses in a variety of animal models [4-8]. However, DNA vaccines alone stimulated only weak T-cell responses in monkeys [9] and humans [10]. To enhance antigen expression levels, various viral vector vaccines have been explored. For example, antigens expressing viral vectors based on poxviruses or adenoviruses were shown to be potent inducers of antigen-specific immune responses in SIV/HIV vaccine studies [9,11]. In addition to increased expression levels, the triggering of innate immune responses by the viral vector particles also seems to contribute to the immunogenicity of viral vector vaccines. However, in contrast to DNA vaccines, repeated immunizations with the same viral vector vaccine appear to be limited by immune responses to the viral vector particles [12,13]. Thus, DNA prime viral vector boost regimens are considered to be one of the most promising strategies to induce long-lasting CTL responses in humans [9,14,15].

In addition to prime-boost regimens, a variety of adjuvants including immunomodulatory cytokines such as GM-CSF [16-22] were explored to improve the efficacy of DNA vaccines.

GM-CSF expression plasmids were co-injected with plasmids encoding vaccine antigens to examine the adjuvant activity in mouse models for HIV-1 [17-19], Hepatitis C virus [20,21] and HSV-2 [22] infection. Coexpression of GM-CSF enhanced antigen-specific T-cell proliferation and humoral immune responses, but had little effect on CTL responses. Over-expression of GM-CSF at the injection site led to the increased recruitment of macrophages and dendritic cells (DCs) [23,24] and influenced the activation status of antigen-presenting cells (APCs) [25]. The temporal and spatial co-expression of antigens and GM-CSF seems to be critical for optimal T-cell priming [26]. In addition, the fusion proteins of antigens and GM-CSF [27] and DNA vaccines encoding such fusion proteins [28,29] were shown to improve antigen-specific antibody responses and cancer immunotherapy. The covalent linkage of the antigen and GM-CSF might allow the targeting of APCs expressing GM-CSF receptors, such as DCs. This could improve antigen uptake and presentation and thus also enhance CD8 T cell responses, similar to targeting strategies based on the macrophage mannose receptor or the DEC205 receptor [30,31]. Therefore, we compared the antigen-specific CD8 T-cell responses induced by DNA vaccines encoding GM-CSF ovalbumin fusion proteins (GM-OVA) with those raised by DNA vaccines coexpressing GM-CSF and ovalbumin (OVA) as two unlinked proteins. Since the antigen expression levels of, and the innate response to, DNA and viral vector vaccines differ considerably, the effect of GM-CSF was determined in DNA immunizations and DNA prime adenoviral vector boost regimens. Surprisingly, immunization with genetic vaccines encoding the GM-CSF ovalbumin fusion protein suppressed CD8+ T cell responses, while coexpression of GM-CSF was found to be a potent stimulator of antigen-specific CD8+ T cell responses. Induction of autoantibodies neutralizing GM-CSF by genetic vaccines encoding the fusion-protein, but not those coexpressing GM-CSF and OVA, might explain the varying effects observed.

## Results

### DNA and adenoviral vector vaccines

**Figure 1:**
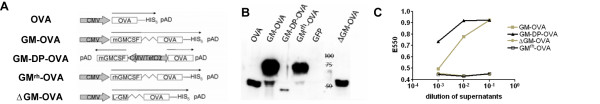
**Map and characterization of vaccine constructs**. (A) Expression cassettes of the indicated vaccine constructs are shown. All open reading frames are under the control of the human cytomegalovirus immediate early promoter (CMV) or its derivative (CMVtetO2). His_6_: C-terminal tag; L-GM: leader peptide of GM-CSF; m: murine; rh: rhesus monkey; "": [Gly4Ser]^3^-linker. (B) Ovalbumin-specific Western Blot analysis of supernatants of 293T cells transiently transfected with plasmids containing the indicated expression cassettes. GFP: supernatant of cells transfected with a GFP expression plasmid. (C) GM-CSF dependent FDCP-1 proliferation. 293 cells were infected with adenoviral vectors containing the indicated expression cassettes and the GM-CSF levels in the supernatants were determined 24 h post infection. Serial dilutions of the supernatants were co-cultured with FDCP-1 cells for 2 days. Proliferation was measured by a MTT assay.

The expression plasmid encoding the fusion protein of GM-CSF and ovalbumin (GM-OVA) was generated by cloning the murine GM-CSF cDNA, a flexible linker, the OVA cDNA and a HIS_6_-tag as a single open reading frame into pcDNA3.1 (Fig. 1A). Three control plasmids expressing ovalbumin alone (OVA), ovalbumin fused to the leader peptide of GM-CSF (ΔGM-OVA), or ovalbumin fused to the open reading frame of rhesus monkey GM-CSF (GM^rh^-OVA), which is biologically inactive in murine cells, were also constructed. Coexpression of murine GM-CSF and OVA from the same plasmid was achieved by a modified bidirectional promoter based on the immediate early gene of human cytomegalovirus (GM-DP-OVA). After transient transfection into 293T cells, expression of the recombinant proteins was detected in cell supernatants by Western Blot analysis. The OVA plasmid and the ΔGM-OVA plasmid expressed a protein of 50 kD, which corresponds to the size of ovalbumin, whereas both fusion proteins migrate at a size of about 75 kD (Fig 1B). The expression levels of the plasmids encoding the fusion proteins were slightly higher than those obtained with OVA and ΔGM-OVA, whereas the expression of ovalbumin from the GM-DP-OVA plasmid is reduced substantially, probably due to a lower activity of the bidirectional promoter. GM-DP-OVA lacks the C-terminal tag explaining the slightly lower size of the protein in the Western blot (Fig. 1B). For further *in vitro *characterization of GM-OVA, the protein was purified from transfection supernatants via Ni^2+^-NTA agarose beads. Using a GM-CSF-dependent cell line, comparable bioactivity was observed for GM-OVA and control supernatants from a GM-CSF producer cell line (data not shown). Adenoviral vectors containing the expression cassettes GM-OVA, GM-DP-OVA, GM^rh^-OVA or ΔGM-OVA were constructed using the pAd-Easy system. Expression of the transgenes and GM-CSF bioactivity was confirmed for the adenoviral vectors after infection of 293 cells. The relative expression levels of OVA by the different adenoviral vector constructs mirrored those observed after transient transfections of the corresponding plasmid DNAs (data not shown). To compare the expression levels of GM-CSF, serial dilutions of the supernatants of 293 cells infected with the adenoviral vectors were tested in a proliferation assay with a GM-CSF-dependent cell line (Fig 1C.) In contrast to the OVA expression levels, the bicistronic vector expressed slightly higher levels of bioactive GM-CSF in comparison to the vector encoding the GM-OVA fusion protein. The supernatants of cells infected with adenoviral vectors enoding GM^rh^-OVA or ΔGM-OVA did not induce proliferation of the GM-CSF-dependent murine cell line. Since GM^rh^-OVA has GM-CSF activity on human cells (data not shown), rhesus monkey GM-CSF is not bioactive on murine cells.

### Influence of the GM-CSF fusion protein on dendritic cell differentiation and antigen presentation

**Figure 2:**
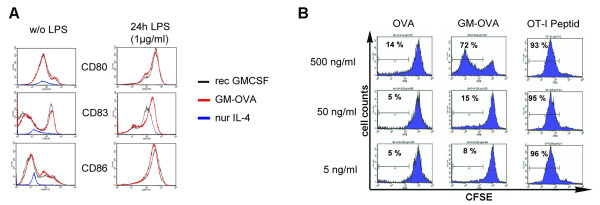
**Influence of GM-OVA on the generation of DC (A) and stimulation of OT-I specific T-cells (B)**. A) BM-derived monocytes were cultured for 8 days in the presence of GM-OVA or recombinant GM-CSF (5 ng/ml each) and IL-4 (1 ng/ml). The CD11c+ cells were analysed for the expression of CD80, CD83, and CD86 surface markers before (left panel) and after addition of LPS (right panel). B) Splenocytes of TCR-transgenic OT-I mice were labelled with CFSE and incubated for 72 h in the presence of either ovalbumin, GM-OVA or OT-I peptide at the indicated final concentration. The addition of recombinant GM-CSF and ovalbumin as separate proteins did not induce proliferation (data not shown).

GM-CSF plays a critical role in the differentiation of dendritic cells. Bone marrow derived monocytes (BMDM) can be differentiated into immature DCs by co-culture with GM-CSF and IL-4. The ability of purified GM-OVA to generate DCs was therefore compared to recombinant GM-CSF. Recombinant GM-CSF and GM-OVA were equally efficient in generating immature DC (low CD80, CD83, CD86) from BMDMs. To a similar extent, the immature DCs induced by GM-OVA or recombinant GM-CSF could be matured by LPS as evidenced by the upregulation of co-stimulatory molecules (Fig 2A). To test whether the fusion of GM-CSF to ovalbumin enhances the activation of antigen-specific CD8+ T cells by antigen-presenting cells (APC), splenocytes from T-cell receptor transgenic OT-I mice were labeled with CFSE and cultured in the presence of decreasing concentrations of GM-OVA or ovalbumin for four days. The proliferation-dependent decrease in CFSE fluorescence of CD8+ T-cells was analyzed by FACS. GM-OVA induced proliferation of OT-I cells at 10-fold lower concentrations than ovalbumin (Fig 2B). The addition of recombinant GM-CSF to ovalbumin-containing cultures did not enhance proliferation of OT-I cells (data not shown), suggesting that the fusion protein enhances presentation of ovalbumin peptides most likely by GM-CSF receptor positive APCs.

### CD8+ T cell responses after immunization with gene-based vaccines encoding the GM-CSF fusion protein

**Figure 3:**
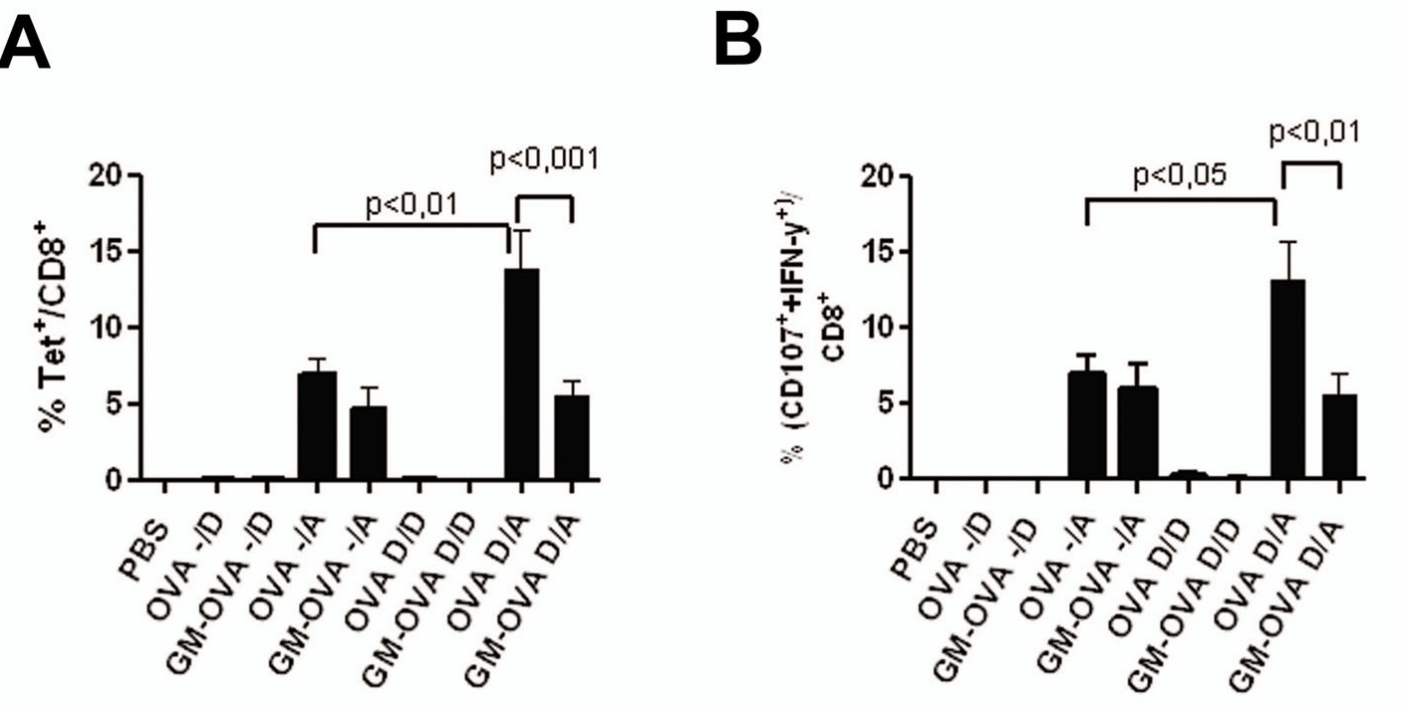
**Prime-boost regimens of GM-OVA suppress OVA-specific CD8+ immune responses**. Mice were immunized by one DNA (-/D), one adenoviral vector (-/A), two DNA (D/D), or a DNA prime adenoviral vector boost (D/A) injection. The DNA and adenoviral vector either encoded ovalbumin (OVA) or the GM-CSF-ovalbumin fusion protein (GM-OVA). One week after a single injection or the second injection the percentage of OT-I tetramer positive CD8^+ ^cells (A) and IFN-γ and CD107a double-positive CD8+ cells after stimulation with the OT-I peptide (B) were determined. All percentages are percent of CD8+ lymphocytes. Mean percentages with SEM of three independent experiments each with three animals per group are shown. Only statistically significant differences between immunization regimens expressing ovalbumin or GM-OVA in the one-way ANOVA test are indicated.

Since the GM-OVA fusion protein maintained GM-CSF bioactivity and enhanced the proliferation of OVA-specific CD8+ T cells in cell culture, we expressed the GM-OVA fusion protein by DNA and adenoviral vectors *in vivo *and determined ovalbumin-specific CD8+ T cell responses. C57BL6 mice were either immunized by one or two (week 0 and 5) DNA injections (-/D and D/D), a single adenoviral vector injection (-/A) or a DNA prime (week 0) and adenoviral vector boost (week 5) regimen (D/A). The DNA and adenoviral vectors either encoded the GM-OVA fusion protein or ovalbumin. One week after the first or second immunization, OVA-specific CD8+ T cell responses were determined using SIINFEKL/H2-K^b ^tetramers. The functional activity of OVA-specific CD8+ T cells was assessed by stimulation with the SIINFEKL peptide followed by staining for interferon-γ and the degranulation marker CD107a [32]. A single injection of DNA encoding either OVA or GM-OVA did not result in any detectable CD8+ T cell responses, while a single adenoviral vector immunization with either antigen resulted in similar levels of tetramer-positive and IFN-γ/CD107a double positive CD8+ T cells, with approximately 6% of the total CD8+ T cells being specific for the SIINFEKL peptide. After two injections of the OVA DNA vaccine, OVA-specific CD8+ T cells became detectable constituting approximately 0.4% of the CD8+ T cells. Two injections of GM-OVA DNA did not result in detectable levels (> 0.1%) of OVA-specific CD8+ T cells demonstrating reduced CD8+ T cell responses for the GM-CSF ovalbumin fusion protein. Significantly reduced CD8+ T cell responses could also be observed for the GM-OVA fusion protein in the DNA prime adenoviral vector boost immunization. The mean percentage of tetramer positive CD8+ T-cells decreased to 5,5% in the GM-OVA group compared to 13,8% of tetramer positive cells in animals immunized with the vaccines only expressing ovalbumin (Fig 3A). Results for the CD107a/IFN-γ staining paralleled the results obtained with the tetramers (Fig. 3B). Priming with the OVA DNA vaccine clearly enhanced CD8+ T cell responses after the adenoviral vector immunization. In contrast, CD8+ T cell responses after the adenoviral GM-OVA boost were not enhanced by priming with GM-OVA DNA (Fig 3A, B).

### Humoral immune responses after immunization with gene-based vaccines encoding the GM-CSF fusion protein

**Figure 4:**
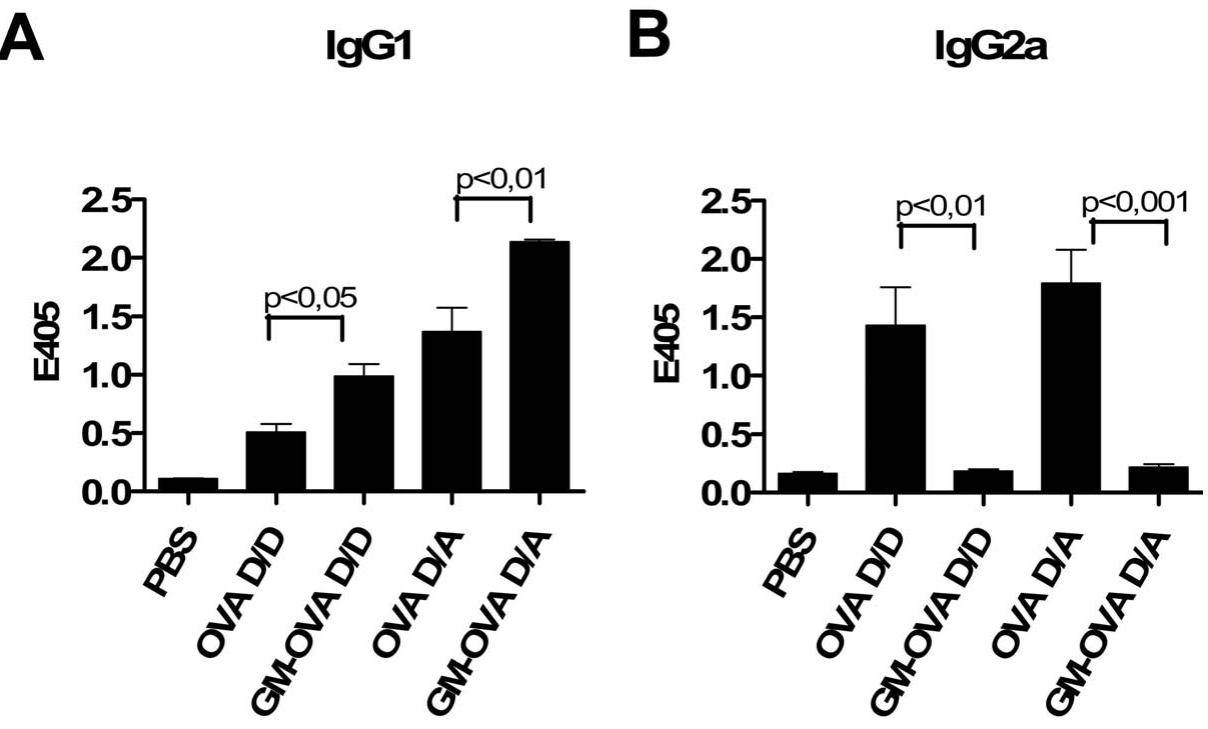
**GM-OVA alters the humoral immune response**. Mice were immunized as described in figure legend 3. Ovalbumin-specific IgG1 (A) and IgG2a (B) antibodies were determined by ELISA. Shown are the mean OD_405 _values with their SEM (n = 6) from the vaccinated animals. Immune sera were diluted 1 to 10 and 1 to 1000 for the determination of IgG2a and IgG1 antibody levels, respectively. Only statistically significant differences between immunization regimens expressing either ovalbumin or GM-OVA in the one-way ANOVA test are indicated.

Since the fusion of GM-CSF to antigens has previously been shown to enhance humoral immune responses to the antigens, we also studied the effect of GM-OVA on OVA-specific IgG1 and IgG2a antibody levels. Since a single injection of the DNA and adenoviral vector vaccines encoding either GM-OVA or OVA did not result in detectable antibody levels, only the results for two repeated DNA injections and the DNA prime adenoviral vector boost regimen are shown (Fig. 4). Strikingly, mice immunized with GM-OVA by DNA or DNA plus adenoviral boost did not produce any detectable levels of ovalbumin-specific IgG2a antibodies. In contrast, immunization with vaccines encoding only ovalbumin resulted in readily detectable levels of antigen-specific IgG2a antibodies (Fig. 4B). Opposite effects were observed on ovalbumin-specific IgG1 antibody levels. These were significantly elevated for the GM-OVA groups in comparison to the groups immunized with the ovalbumin-expressing vaccines (Fig. 4A).

### Stimulation of CD8+ T cell responses by vaccines coexpressing GM-CSF and ovalbumin

**Figure 5:**
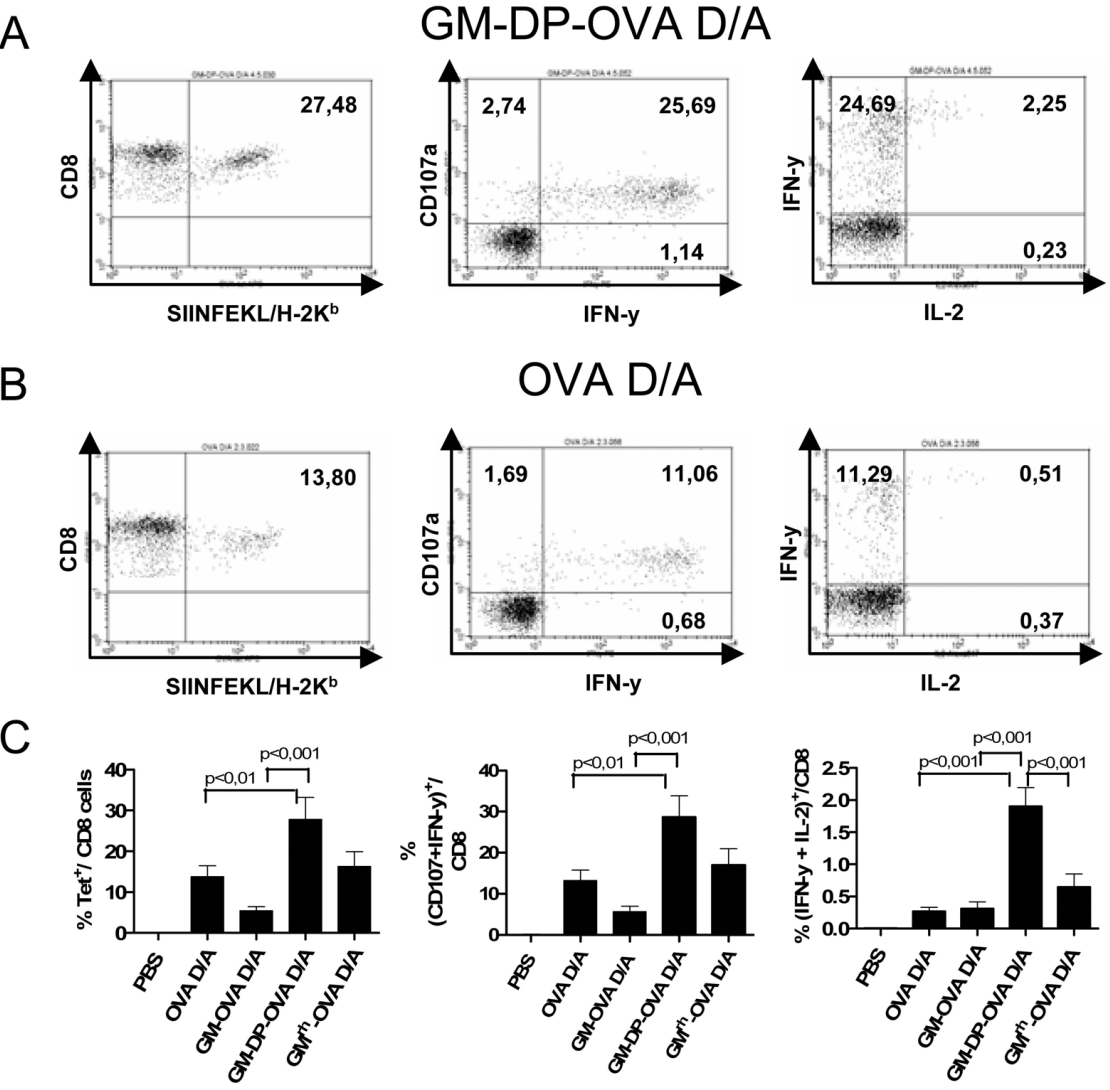
**Coexpression of GM-CSF and ovalbumin enhances CD8+ immune responses**. Mice were immunized by the DNA prime adenoviral vector boost as described in figure legend 3 with the DNA and adenoviral vectors encoding the indicated expression cassettes (s. Fig. 1A). Representative dot blots for SIINFEKL tetramer staining (left column), CD107 and IFN-γ staining (center column) and IFN-γ and IL-2 staining (right column) of CD8+ lymphocytes obtained from individual mice immunized with GM-DP-OVA (A) and OVA (B) are shown. Panel C gives the mean and SEM for the immunization groups indicated and the parameters analysed. Only statistically significant differences between the groups are indicated. Three independent immunization experiments with three animals per group were performed for the PBS, OVA, and GM-OVA groups, while one experiment was performed with a total of 6 mice for the GM-DP-OVA and the GM^rh^-OVA groups.

Suppression of CD8+ T cell responses by the GM-OVA vaccines could be directly due to the bioactivity of GM-CSF. If this were the case, the coexpression of GM-CSF and ovalbumin from bicistronic expression cassettes should also suppress CD8+ T cell responses. However, the opposite effect was observed. Despite substantially lower ovalbumin expression levels by the GM-DP-OVA vaccines coexpressing GM-CSF and ovalbumin, DNA prime adenoviral vector boost immunization with GM-DP-OVA vaccines enhanced the percentage of SIINFEKL-specific CD8+ T cells compared to vaccines only expressing ovalbumin (Fig 5, left column). This is in sharp contrast to the results obtained after GM-OVA vaccination, which reduced the percentage of tetramer positive CD8+ cells.

**Figure 6:**
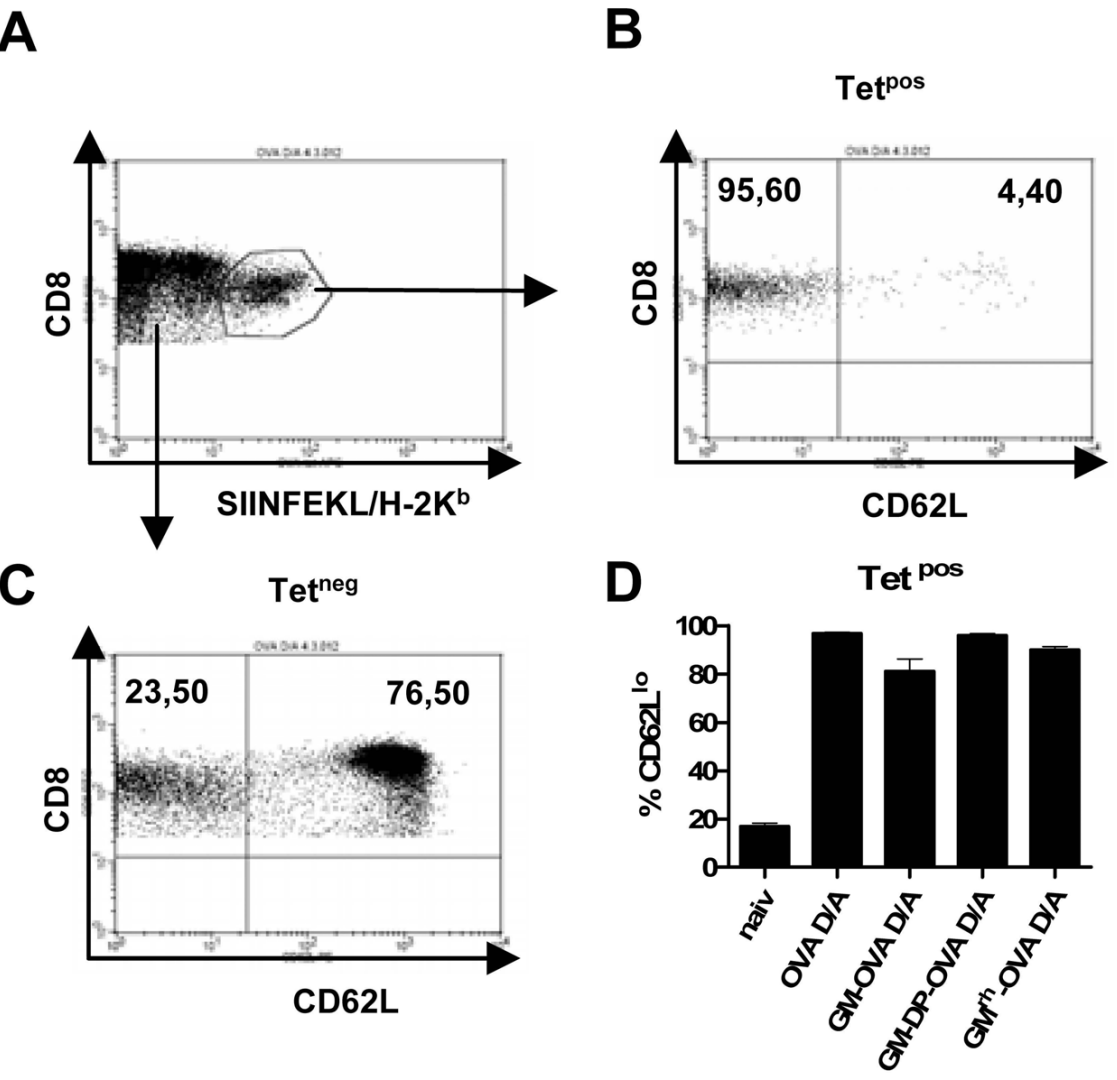
**GM-CSF does not influence the activation status of CD8+ lymphocytes**. Mice were immunized as described in figure legend 5. The effector phenotype of the antigen-specific CD8+ cells was confirmed by costaining for CD8, SIINFEKL/H-2K^b^, and CD62L. CD8 and Tetramer double positive cells were gated (A) and analysed for CD62L expression (B, D). D) Mean percentage and SEM of CD62L-low cells from the CD8 and Tetramer double positive cells from three mice per group are shown. Panel C gives a representative example of CD62L expression levels of the CD8+, but tetramer negative population.

After immunization with the bicistronic vaccines more than a quarter of all CD8+ cells were specific for ovalbumin. Given this extraordinarily high CD8+ T cell response, the OVA-specific cells were further characterized functionally. Stimulation of the spleen cells of mice immunized with the bicistronic vaccine with the SIINFEKL peptide also resulted in more than 25% IFN-γ positive CD8+ cells. Most of IFN-γ positive cells also displayed cytotoxic activity as indicated by costaining with the CD107a degranulation marker (Fig 5, center column). In addition, the coexpression of GM-CSF enhanced the percentage of IFN-γ and interleukin 2 double-positive cells after peptide stimulation, confirming the polyfunctional nature of the CD8+ T cell responses induced by this potent immunization regimen (Fig 5, right column). About 90% of all tetramer-positive CD8 cells also showed the downregulation of CD62L expression (Fig 6), which further indicates an effector phenotype [33,34]. No qualitative differences among the various immunization groups were detectable, suggesting that GM-CSF only influences the quantity but not the functional properties of the vaccine-induced CD8+ T-cells.

### CD8 T cell responses induced by vaccines encoding a biologically inactive GM-CSF ovalbumin fusion protein

In contrast to the GM-OVA vaccines, the coexpression of GM-CSF and ovalbumin did not suppress CD8 T cell responses. This indicates that the suppressive effects of GM-OVA vaccines on the CD8 T cell responses are not simply due to the biological activity of GM-CSF. To exclude the possibility that immune responses were affected by altered antigen expression levels, subcellular localization, and/or stability of the fusion protein, mice were also immunized with DNA and adenoviral vector vaccines expressing a fusion protein of rhesus macaque GM-CSF and ovalbumin. Although fully active on primate cells, rhesus monkey GM-CSF was inactive in rodent cells (Fig. 1C). In all parameters investigated, including Tetramer analyses and intracellular staining for IFN-γ, interleukin 2, and/or CD107a, the immune response induced by the rhesus monkey GM-OVA vaccines did not differ significantly from the response induced by the OVA vaccines (Fig. 5 and 6).

### Antibody responses induced by gene-based vaccines encoding bioinactive GM-CSF fusion protein or coexpressing GM-CSF and ovalbumin

**Figure 7:**
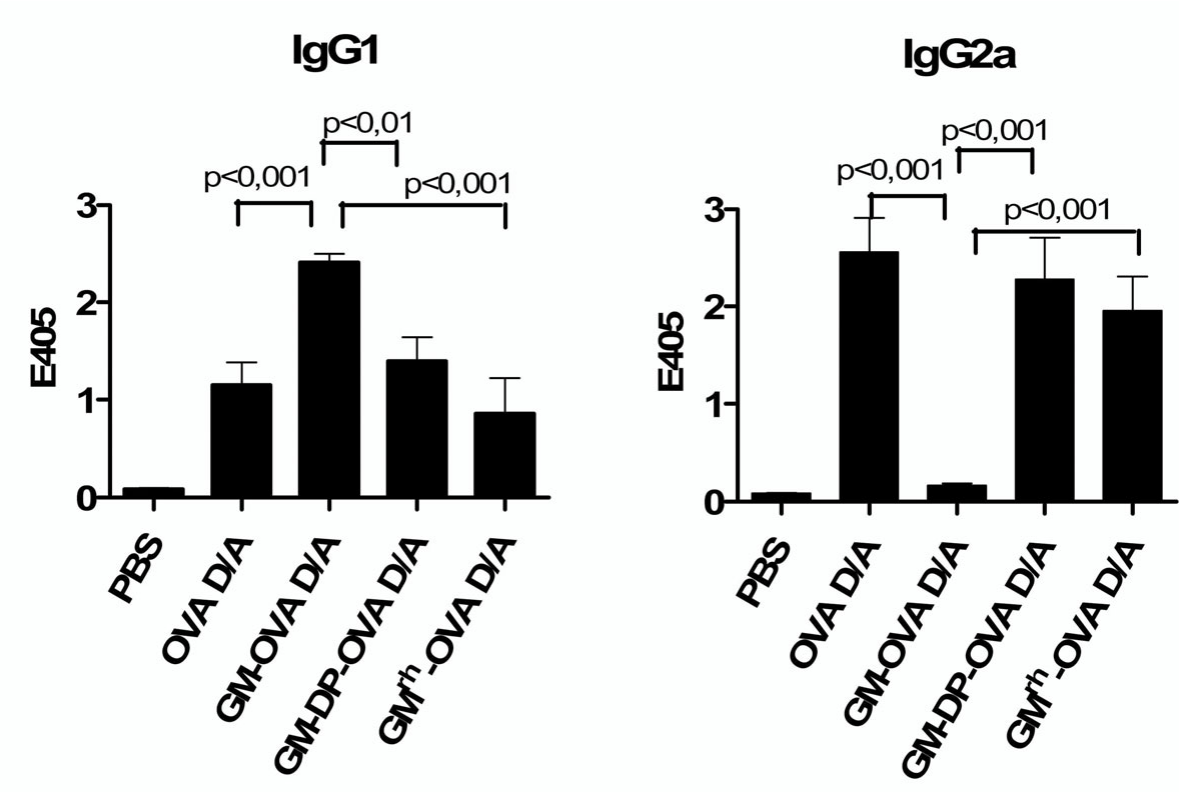
**Coexpression of GM-CSF and ovalbumin does not bias the humoral immune response**. Mice were immunized by DNA prime adenoviral vector boost injections as described in figure legend 5. Ovalbumin-specific IgG1 (A) and IgG2a (B) antibodies were determined by ELISA. Shown are the mean OD_405 _values with their SEM (n = 6) from the vaccinated animals. Immune sera were diluted 1 to 10 and 1 to 1000 for determination of IgG2a and IgG1 antibody levels, respectively. Only statistically significant differences between immunization regimens expressing either ovalbumin or GM-OVA in the one-way ANOVA test are indicated.

The GM-OVA vaccines not only suppressed cellular immune responses but also the antigen-specific IgG2a antibody response (Fig 4B). Therefore, ovalbumin-specific antibody levels of the IgG1 and IgG2a subtype were also determined for the vaccines coexpressing GM-CSF and ovalbumin or expressing the fusion protein of inactive rhesus GM-CSF and ovalbumin. Vaccinations with GM-OVA again suppressed OVA specific IgG2a antibody responses in comparison to the vaccine expressing only ovalbumin (Fig. 7). In contrast, the coexpression of GM-CSF or the expression of the fusion proteins of rhesus monkey GM-CSF and ovalbumin did not affect IgG1 and IgG2a antibody levels in the DNA prime adenoviral vector boost immunization regimen.

### Induction of neutralizing antibodies to GM-CSF

**Figure 8:**
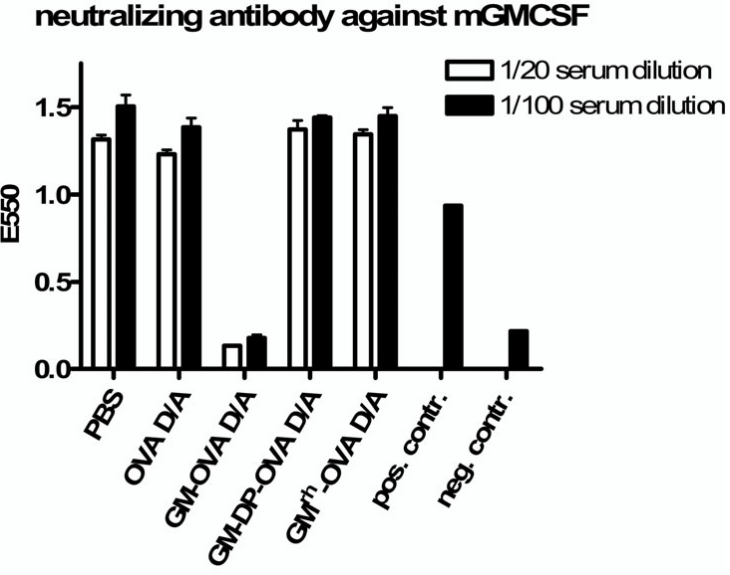
**GM-OVA induces neutralizing antibodies against murine GM-CSF**. Mice were immunized with the indicated DNA prime adenoviral vector boost regimens. One week after the adenoviral vector booster immunization, the GM-CSF neutralizing activity was determined on the GM-CSF dependent FDCP-1 cell line stimulated with 2,5 ng murine GM-CSF/ml). Mouse sera were tested in a 1/20 (white bars) and a 1/100 (black bars) dilution. Medium without GM-CSF served as a negative control, whereas GM-CSF-containing medium without mouse serum was the positive control.

Whereas CD8 T cell responses upon injection of the Ad-OVA vaccine were significantly increased in DNA-OVA primed mice, such a priming effect was not observed with the DNA-GM-OVA prime and Ad-GM-OVA boost regimen (Fig. 3), suggesting that immune responses elicited by priming with DNA-GM-OVA might have suppressed the secondary response. Since neutralizing antibodies to GM-CSF have been observed previously after injection of recombinant GM-CSF proteins [35,36], we analysed sera from immunized mice for the presence of GM-CSF neutralizing antibodies using the GM-CSF-dependent FDCP-1 cells. While the sera of mice immunized with the GM-OVA vaccines inhibited GM-CSF-dependent cell growth down to a 1:100 dilution, no inhibitory activity on FDCP-1 cells was observed for the sera of mice immunized with vaccines coexpressing GM-CSF and OVA or expressing the monkey GM-CSF ovalbumin fusion proteins (Fig. 8). Consistently, autoantibodies binding to GM-CSF in an ELISA were only detected in mice immunized with vaccines encoding the fusion proteins, but not in any of the other vaccine groups (data not shown).

### Influence of neutralizing antibodies to GM-CSF on immune responses and viral replication in a retroviral infection model

**Figure 9:**
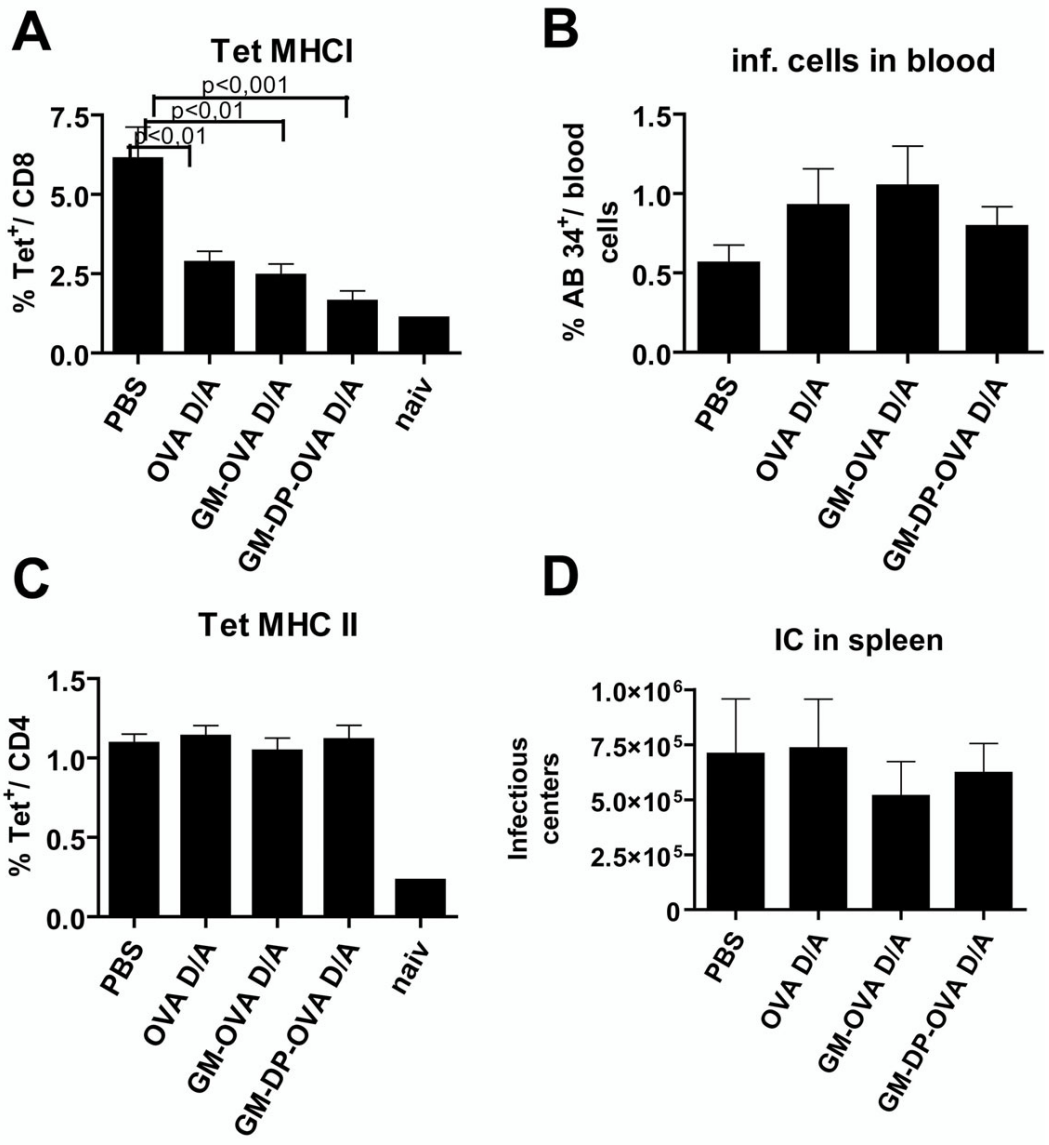
**GM-CSF autoantibodies do not interfere with the control of FV infection**. GM-CSF autoantibodies were induced by DNA prime adenoviral vector boost immunization with the GM-OVA vaccines. As controls, groups of mice were immunized with the OVA or the GM-DP-OVA vaccines, or were mock vaccinated (PBS). Two weeks after the adenoviral vector booster immunization, mice were challenged with 3000 SFFU of FV. 11 days post challenge FV-specific immune responses were monitored by MHC I (A) and MHC II (C) tetramer staining. Mean percentages and SEM (n = 6) of Tet^+ ^CD8^+ ^(A) and CD4^+ ^(C) cells are shown. Splenocytes of an uninfected animal were used as negative control (naiv). The viral load in the blood was detected by AB34^+ ^cells via flow cytometry (B). In addition the numbers of infectious centers per spleen were determined (D).

Since GM-CSF knock-out mice had an impaired CD8+ T cell response after immunization with peptides [37], we determined the relevance of neutralizing anti-GM-CSF antibodies induced by immunization with the GM-OVA vaccine, in the Friend-Virus (FV) infection model. Immunological control of FV infection was shown to be dependent on antibodies and CD4+, and CD8+ T-cell responses [38,39]. To induce neutralizing GM-CSF antibodies mice were primed with the DNA and boosted with the adenoviral vector vaccine, both expressing GM-OVA. As controls, mice were immunized against OVA with vaccines expressing either ovalbumin alone or GM-CSF and ovalbumin from a bicistronic expression cassette. One week after the adenoviral vector boost, neutralizing antibodies were detected in all animals immunized with the GM-OVA vaccines, but not in any of the other groups. The antibodies remained detectable during the whole observation period (data not shown). Induction of virus-specific CD8+ and CD4+ T cell responses was determined by MHC-I and MHC-II tetramer staining 11 days post challenge with FV. Similar levels of antigen-specific CD4+ and CD8+ T cell responses were observed in all immunized groups (Fig. 9A,C). In addition, FV loads in the blood and the spleen were not affected notably by the type of vaccine antigen (Fig. 9B,D). Thus vaccine-induced neutralizing antibodies against GM-CSF neither influenced T cell responses nor viral load after FV infection. Interestingly, all immunized groups had significantly reduced numbers of FV-specific CD8^+^-T-cells compared to the non-immunized control animals (Fig. 9A), suggesting some interference between vaccine-induced immune responses and antiviral CD8+ T cell responses after FV infection.

## Discussion

A side-by-side comparison of a DNA prime adenoviral vector boost immunization regimen leading either to the expression of a fusion protein of GM-CSF and the model antigen or to the coexpression of GM-CSF and antigens as two separate proteins revealed striking differences in the induction of polyfunctional antigen-specific CD8+ T cell responses and humoral immune responses. Immunization with gene-based vaccines encoding the bioactive GM-CSF antigen fusion proteins suppressed CD8+ T cell responses, while the coexpression of GM-CSF and antigens stimulated these responses. Although the fusion proteins enhanced the stimulation of antigen-specific CD8+ T cells in coculture experiments with antigen-presenting cells, the induction of neutralizing antibodies to GM-CSF probably counteracted any beneficial effect of the fusion protein on the enhancement of antigen uptake or presentation *in vivo*. the injection of recombinant GM-CSF proteins or GM-CSF fusion proteins has been shown previously to induce antibodies to GM-CSF in mice and humans [29,35,36,40]. Differences in postranslational modifications such as glycosylation patterns between the recombinant proteins and the endogenously expressed GM-CSF have been postulated to be responsible for this loss of tolerance [35,40]. Since the GM-CSF expression levels after infection with the bicistronic vector was the same or even higher when compared to the GM-OVA encoding vector, we can exclude that simple overexpression of the cytokine is a reason for the break of tolerance. However, the induction of GM-CSF neutralizing antibodies by genetic vaccines expressing GM-CSF antigen fusion proteins in the vaccinees suggests an alternative mechanism for this autoimmune response. B-cells with antigen receptor specificities for GM-CSF might take up the GM-CSF-antigen fusion protein and present antigen-derived epitopes on MHC-II molecules. T-helper cells specific for the antigen part of the GM-CSF fusion protein could then stimulate these B-cells even in the absence of autoreactive GM-CSF-specific T-helper cells. This would be consistent with a previous hypothesis for the appearance of autoreactive antibodies [41] and with results from immunization studies with other fusion proteins [42,43].

The consequences of neutralizing GM-CSF antibodies for the host are not well defined. Reconstitution of white blood cells after bone-marrow transplantation and immune responses to a protein vaccine were not impaired in the presence of GM-CSF autoantibodies [44]. Using the FV infection model, we did not observe any negative effects associated with GM-CSF autoantibodies either. Consistently, no pathological alterations have been reported to be associated with anti-GM-CSF antibodies in cancer patients. However, GM-CSF knock out mice were not able to generate CD8+ T cell responses after peptide immunization and IgG2a antibody production was also reported to be delayed after protein immunization [37]. In the present study, induction of GM-CSF neutralizing antibodies coincided with suppression of CD8+ T cell responses induced by a DNA prime adenoviral vector boost regimen. Lack of suppression of CD8+ T cell responses after a single immunization with the same DNA or adenoviral vector vaccine further supports a causal relationship between GM-CSF antibodies and suppression of CD8+ T cell responses, since in these immunization experiments GM-CSF neutralizing antibodies are absent at the time point of T cell priming. Depending on the precise immunostimulatory pathways employed by a vaccine or pathogen, the requirement for GM-CSF might differ significantly, providing an explanation for the different observations made in the mouse models. Although there is no direct evidence of clinical complications so far, possible long-term consequences of the anti-GM-CSF responses, such as involvement in pulmonary alveolar proteinosis (PAP) [45,46], are difficult to exclude. Given the loss of tolerance by other fusion proteins [42,43] and the immunogenicity of genetic vaccines, a more general note of caution regarding the use of genetic vaccines encoding fusion proteins between host proteins and heterologous antigens might even be justified.

In contrast to immunization with genetic vaccines encoding the GM-CSF-antigen fusion proteins, which strongly suppressed IgG2a antibody responses, the coexpression of GM-CSF and antigens in the DNA prime adenoviral vector boost regimen did not modulate IgG1 and IgG2a antibody responses notably. This was unexpected, since repeated immunizations with adenoviral vectors coexpressing GM-CSF and the amyloid-beta protein by the intranasal route resulted in a Th2-type immune response [47]. In addition, the coexpression of GM-CSF and antigens in numerous DNA immunization studies increased antibody responses [16,18,20-22]. Since the bicistronic expression cassette in our study expresses lower amounts of ovalbumin than the vaccine encoding ovalbumin only, GM-CSF coexpression seems to compensate for the lower antigen expression levels in the sense that in the presence of GM-CSF a lower amount of antigen is needed to induce comparable humoral immune responses than in its absence. Previous studies also show that increased antigen levels correlate with increased antibody titers, but do not influence the dominant immunoglobulin subtype[48]. Thus, the difference in the IgG1/IgG2a ratio observed after immunization with the genetic vaccine encoding the GM-CSF-Ova fusion protein does not seem to be simply due to GM-CSF receptor signalling, since this also occurs by the coexpression of GM-CSF. However, immunization with vaccines encoding rhesus monkey GM-CSF fused to ovalbumin did not lead to a change in the IgG1/IgG2a ratio either, although no differences were observed between the expression levels of the two GM-CSF fusion proteins. This indicates that the change in the antigen-specific IgG1/IgG2a ratio observed after immunization with the fusion protein depends on the GM-CSF bioactivity of the fusion protein and the covalent linkage of GM-CSF with the antigen.

The immunomodulatory properties of GM-CSF could also differ substantially with the type of antigens, the route of immunization, and/or the type of genetic vaccine used. This also seems to apply to the effect of GM-CSF on CD8+ T cell responses. Coexpression of GM-CSF and antigens by DNA vaccines was shown to stimulate CD8+ T cell responses in some studies [17,49] but not in others [19,50]. In the context of a DNA prime adenoviral vector boost regimen, we observed that GM-CSF was a strong stimulator of CD8+ T cell responses. Coexpression of GM-CSF by the DNA and adenoviral vector vaccines enhanced the percentage of CD8+ T cells specific for the immunodominant epitope of the antigens from approximately 11% to more than 25% as determined by tetramer staining and intracellular IFN-γ staining. Although we can not completely rule out that the different antigen expression levels influence the strength of immune response, it seems unlike that lower antigen levels induce stronger CTL responses. Rather, it has been reported that increased antigen expression levels obtained by codon-optimization or enhanced promotor activities correlate with the better induction of IFN-γ producing T-cells or cytotoxic T-cells [48,51].

Coexpression of GM-CSF and ovalbumin by DNA prime adenoviral vector boost immunizations led to a strong CD8+ T cell response, with more than a quarter of all CD8+ T cells being specific for a single immunodominant peptide. Although the percentage of CD8+ T cell specific for other antigens might well be lower than those obtained in the present study with the ovalbumin model antigens, the enhancement of CD8+ T cells responses by GM-CSF is encouraging. The antigen-specific CD8+ T cells induced in the presence of GM-CSF were not only numerous, but also displayed markers of polyfunctional T cells such as the coexpression of IFN-γ, IL-2, and the CD107a degranulation marker. T cells that produce both IFN-γ and IL-2 were recently proposed to be important in the control of chronic viral infections, like CMV, EBV or in HIV LTNP and might be indicative of long-lived memory T-cells [52]. Although the functional relevance of the CD8+ T cell response induced by DNA and adenoviral vector vaccines coexpressing GM-CSF and antigens needs to be confirmed in relevant tumor and infection models, GM-CSF should be considered as a CD8+ T-cell adjuvant in DNA prime adenoviral vector immunization regimens.

## Conclusion

Genetic vaccines encoding fusion proteins between antigens and a host protein led to the rapid induction of autoantibodies. Therefore a more general note of caution regarding the use of highly immunogenic viral vector vaccines encoding such fusion proteins seems to be justified. In contrast, the coexpression of GM-CSF and antigens as two separate proteins in DNA prime adenoviral vector immunization regimens led to the enhanced induction of polyfunctional CD8+ T-cells, further supporting a potential application of GM-CSF as an adjuvant not only for DNA, but also for viral vector vaccines. Given the fact that the DNA prime adenoviral vector boost regimen is presently one of the most efficient ways to induce CD8+ T cell responses in mice, non-human primates and humans, the enhancement of this response by GM-CSF is a striking observation.

## Methods

### DNA and adenoviral vector vaccines

The DNA constructs used for the immunization studies are all based on the expression plasmid pcDNA3.1 (Invitrogen, Karlsruhe, Germany) or pShuttle-CMV [53]. The coding sequence of murine GM-CSF was amplified by RT-PCR from RNA isolated from stimulated mouse splenocytes and cloned into the pCR-2.1-TOPO vector (Invitrogen). All other plasmids were constructed via standard cloning techniques including overlap extension PCR. Transgene expression is driven in all constructs by the immediate early promoter/enhancer region of human cytomegalovirus. The open reading frame of the fusion protein GM-OVA consisting of murine GM-CSF, a [Gly_4_Ser]^3 ^linker and OVA was cloned into the pcDNA3.1 vector. pOVA, also a pcDNA3.1 derivative, encodes ovalbumin itself. Both proteins have a C-terminal HIS_6_-tag. The plasmids pGM^rh^-OVA, pGM-DP-OVA, and pΔGM-OVA are based on the pShuttle-CMV vector. The GM^rh^-OVA fusion protein is equivalent to the mouse one with the sequence of murine GM-CSF being replaced by the rhesus monkey homologue. In the plasmid pGM-DP-OVA, transgene expression is driven by a bidirectional version of the CMV/TetO2 promoter [54]. In pΔGM-OVA the coding sequence of ovalbumin is preceeded by the 17 amino acid long signal peptide of murine GM-CSF. All DNA preparations for immunizations were carried out with the Endofree Plamid Mega or Giga Kit (Qiagen, Hilden, Germany).

All E1-deleted, replication-defective adenoviruses with the corresponding expression cassettes (Ad-GM-OVA, Ad-GM-DP-OVA, Ad-rhGM-OVA, Ad-ΔGM-OVA) were generated by the AdEasy-system [53]. The pShuttle plasmids and pAdEasy1 were electroporated into BJ5183 bacteria as previously described [54]. Correctly recombined plasmids were transfected into 293 cells. Viral vectors growing out were checked for transgene expression by Western Blot analyses and GM-CSF bioactivity, if applicable. Vector particles were purified by CsCl gradient centrifugation and quantified by optical density measurements. In addition, the TCID_50_of the vectors were determined on 293 cells. The adenoviral vector preparations were also tested for endotoxin levels with the LAL quantification assay (Cambrex Bio Science, Verviers, Belgium), confirming that the dose used for immunization of mice contained less than 0,1 EU.

### Cell culture media and reagents

HEK293 and 293T cells were cultured in DMEM containing 10% FCS and 1% penicillin/streptomycin. RPMI 1640 supplemented with 10% FCS, 2 mM L-Glutamine, 10 mM HEPES, 50 μM β-Mercaptoethanol and 1% antibiotic/antimycotic (all Gibco, Karlsruhe, Germany) was used for the lymphocyte cultures (R10-medium). Bone-marrow cultures were grown in RPMI 1640, supplemented with 10% FCS, 1% penicillin/streptomycin, 4 mM L-Glutamine, 1 mM sodium pyruvate and recombinant mouse IL-4 (1 ng/ml) and GM-CSF (5 ng/ml) (Biomol, Hamburg, Germany).

### Expression and bioactivity of GM-OVA

293T cells were transfected using the calcium/phosphate precipitation method. Supernatants were collected 48 h after transfection and tested for protein expression by Western Blot analysis. A combination of rabbit-α-OVA (Chemicon International, LTD, Hampshire, UK) and goat-α-rabbit-HRP (Sigma, Munich, Germany) antibodies was used for detection. To confirm the bioactivity of GM-OVA, it was purified from supernatants of transfected cells by Ni-NTA-affinity chromatography as described by the manufacturer (Qiagen, Hilden, Germany). The purified protein migrated as a single band of the expected size in Coomassie stained polyacrylamide gels. The GM-CSF-dependent FDCP-1 cells were incubated with the purified GM-OVA or supernatants from NIH 3T3 cells stably expressing GM-CSF. The GM-CSF content of the supernatants had been determined by ELISA. After 48 h, GM-CSF-dependent cell growth was monitored by MTT-assay as described elsewhere [55,56]. Additionally, the ability to generate DCs from bone marrow cultures was tested as previously described [57]. Briefly, bone marrow derived monocytes were cultured in the prescence of IL-4 and recombinant GM-CSF or GM-OVA for 8 days, with or without LPS maturation for 24 h. Their phenotype was characterized by surface staining with the antibodies α-CD11c-APC, α-CD80-FITC, α-CD86-FITC (all BD Bioscience, Heidelberg, Germany) and α-CD83-PE (eBioscience, San Diego, USA) in FACS analyses.

### OT-I proliferation assay

Splenocytes of transgenic OT-I mice were incubated with CFSE (3μM; Molecular Probes, Eugene, Oregon, USA) at a density of 8 × 10^7 ^cells/ml for 6 min at room temperature with gentle mixing. The labelling reaction was stopped by adding one volume of FCS after which the cells were washed twice with PBS. Thereafter, cells were plated into 96-well plates at a density of 1 × 10^6 ^cells/well and incubated with different concentrations of either GM-OVA or OVA (500 to 0,5 ng/ml) for 4 days. After washing the cells twice with PBS containing 0,5% BSA and 1 mM sodium azide (PBS/BSA/Azid), cell proliferation was measured in FACS analysis. Cells, which were incubated with OT-I peptide, were used as positive controls whereas non-stimulated cells served as negative ones.

### Animals and immunizations

6–8 week old female C57BL/6N mice were purchased from Janvier (Le Genest-ST-Isle, France) and housed in singly-ventilated cages in accordance with the national law and institutional guidelines.

All vaccines were diluted in PBS and injected subcutaneously in both hind foot pads. For single dose experiments, mice were immunized on day 1 with either 50 μg of DNA (pOVA or pGM-OVA) or 5 × 10^9 ^adenoviral particles (Ad-ΔGM-OVA resp. Ad-GM-OVA) corresponding to 2 × 10^8 ^50% tissue culture infectious doses (TCID_50_). After one week, serum samples were collected and on day 8 animals were sacrificed to analyze the CTL responses. In the prime-boost experiments, all animals received an additional DNA injection (pOVA or pGM-OVA) 5 weeks prior to the above mentioned protocol. Serum samples were collected 28 days after the first and 7 days after the second immunization, whereas the T-cell assays were carried out 8 days after the second immunization.

### OVA specific antibody ELISA

Blood was taken retro-orbitally and serum was collected after centrifugation for 5 min at 5.000 g in a table top centrifuge. Ovalbumin protein (Sigma) was coated on 96-well plates (MaxiSorb, Nunc, Wiesbaden, Germany) at a final concentration of 5 μg/ml. After blocking with 5% milk powder, serum samples were added at appropriate dilutions and incubated for 1 h, followed by intensive washing. Alkaline phosphatase-coupled antibodies against mouse IgG1 or IgG2a antibodies (BD Bioscience) were added and incubated for 1 h. The enzymatic reaction was developed with the pNPP substrate (Sigma) for 30 min. Reaction was stopped by sodium hydroxide solution (1 M) and the optical densities were measured at a wavelength of 405 nm.

### Tetramer and intracellular cytokine staining (ICS)

Splenocytes were collected at indicated time points. After red blood cell lysis, 1 × 10^6 ^cells were plated in 96-well round-bottom plates (Nunc) for each staining.

For the tetramer staining, cells were washed once and incubated with 2 μl of SIINFEKL/H-2K^b^-APC tetramers (Sanquin, Amsterdam, NL) in total volume of 100 μl PBS/BSA/Azid for 40 min at room temperature. After surface staining with α-CD8-FITC, cells were incubated with 7-amino-actinomycin D (7-AAD) for 5 min to exclude dead cells from subsequent FACS analyses. In some experiments, α-CD62L-PE antibodies were included in the tetramer analysis to characterize the CD8^+ ^activation status.

For ICS, samples were stimulated for 6 h in the presence of 2 μM Monensin, which inhibits the cytokine secretion, and 1 μl α-CD107a-FITC, which is a marker for lymphocyte degranulation [32]. Cells were either stimulated by α-CD3 and α-CD28 antibodies (2 μg/ml and 1 μg/ml, respectively) or the OT-I/SIINFEKL peptide (2 μg/ml, Genaxxon, Biberbach, Germany) and compared to non-stimulated cultures. After stimulation, surface staining was carried out with αCD8-PerCP or αCD4-FITC (BD Bioscience). Cells were fixed in 2% paraformaldehyde, followed by permeabilisation with 0,5% Saponin in PBS/BSA/Azid buffer. Cytokines were detected with αIFN-γ-PE and αIL-2-AlexaFluor647.

### Friend virus challenge and virus detection

Mice were injected intravenously with 0.5 ml phosphate buffered saline (PBS) containing 3,000 spleen focus-forming units (SFFU) of the Friend virus complex (FV). The B-tropic, polycythemia-inducing FV complex used in all experiments was from uncloned virus stocks obtained from 10% spleen cell homogenates as described previously [58].

For infectious center assays, single-cell suspensions from infected mouse spleens were cocultivated with *Mus dunnis *cells at 10-fold dilutions. Cultures were incubated for 5 days, fixed with ethanol, stained with F-MuLV envelope-specific monoclonal antibody 720, and developed with peroxidase-conjugated goat antimouse antibody and aminoethylcarbazol to detect foci. Tetramer analyses were done by flow cytometry as described previously [59]. For the quantification of Friend virus-infected blood cells, single-cell suspensions of nucleated, live cells were analyzed by flow cytometry. To detect Friend virus infection cells were stained as described previously with tissue culture supernatant containing Friend murine leukemia virus glycosylated Gag-specific monoclonal antibody 34 (AB34) [60].

### Statistical analysis

Results are expressed as the means ± standard errors of the means (SEM). Statistical comparisons were performed by one-way ANOVA test, followed by a Bonferroni post test using the Prism 4.0, GraphPad Software. P < 0,05 was considered as statistically significant.

## Authors' contributions

MT participated in the design of the experiments, carried out the main part of the experiments, analyzed the data and wrote the manuscript with KÜ. BT established and performed the antibody ELISA, while SS and NG performed the Friend virus infection experiments. SK participated in the design of the study, adviced in the construction of the adenoviral vectors and provided assitance during the animal experiments. UD supported the study by critical discussions, designed the Friend virus infection experiments and critically reviewed the manuscript. KÜ designed and coordinated the study, contributed to interpretation of the data, and wrote the manuscript with MT. All authors read and approved the final manuscript
